# Prolonged febrile syndrome. What could we find?

**DOI:** 10.1186/1471-2334-14-S7-P57

**Published:** 2014-10-15

**Authors:** Cozmina Andrei, Georgeta Ducu, Daniela Camburu, Elisabeta Benea

**Affiliations:** 1 National Institute for Infectious Diseases "Prof. Dr. Matei Balş", Bucharest, Romania; 2 Carol Davila University of Medicine and Pharmacy, Bucharest, Romania

## Background

Often a patient with prolonged fever requires a complex medical approach involving several medical specialties and extensive laboratory investigations.

## Case report

We report the case of a 32 year male who presented for prolonged febrile syndrome about 2 months, chills and discreet urinary symptoms, initially interpreted to be a chronic prostatitis and treated for about 40 days with fluoroquinolones. His medical history included myasthenia gravis with immunosuppressive treatment and thymectomy. Physical examination revealed patient with fever especially at night, a tumor of 10/5 cm in the right coxofemoral joint region, right testicular swelling, difficulty in urinating.

The biological findings showed lymphopenia, CD4=64 cells/cmm, a marker of sepsis positive, incomplete cholestatic syndrome, urinalysis with leukocytes and erythrocytes, negative urine culture, blood cultures were negative. Genitourinary ultrasound and urography were performed, which found enlarged epididymis with multiple nodules, heterogeneous prostate. Polymerase chain reaction from urinary sediment was positive for *Mycobaterium tuberculosis*.

Given the results of the investigations, urinary tuberculosis was suspected and anti-tuberculous (TB) treatment was initiated while waiting for the culture results. Patient’s clinical status worsened, with the persistence of fever and chills, and the increase of tumor growth in the right coxofemoral joint region. The soft tissue ultrasound showed fluid collection along the psoas muscle straight to the scrotal region. Following surgery the fluid collection from right coxofemoral joint region was drained while the patient continued TB therapy with fever remission.

## Conclusion

In our country tuberculosis is a real public health problem, especially in immunocompromised patients. In literature, extrapulmonary tuberculosis such as genitourinary TB is noted as the second most common form of extrapulmonary TB in countries with increased incidence of tuberculosis. However there are no multicenter studies with a high level of evidence on this problem.

## Consent

Written informed consent was obtained from the patient for publication of this Case report and any accompanying images. A copy of the written consent is available for review by the Editor of this journal.

